# Decoding first complete chloroplast genome of toothbrush tree (*Salvadora persica* L.): insight into genome evolution, sequence divergence and phylogenetic relationship within Brassicales

**DOI:** 10.1186/s12864-021-07626-x

**Published:** 2021-04-30

**Authors:** Abdul Latif Khan, Sajjad Asaf, Lubna, Ahmed Al-Rawahi, Ahmed Al-Harrasi

**Affiliations:** 1 Natural and Medical Sciences Research Center, University of Nizwa, 616 Nizwa, Oman; 2 Department of Botany, Garden Campus, Abdul Wali Khan University, Mardan, 23200 Pakistan

**Keywords:** Salvadoraceae, Sequencing, Repeat analysis, Divergence, Phylogenomics, InDel, SNP, Chloroplast

## Abstract

**Background:**

*Salvadora persica* L. (Toothbrush tree – Miswak; family-Salvadoraceae) grows in the arid-land ecosystem and possesses economic and medicinal importance. The species, genus and the family have no genomic datasets available specifically on chloroplast (cp) genomics and taxonomic evolution. Herein, we have sequenced the complete chloroplast genome of *S. persica* for the first time and compared it with 11 related specie’s cp genomes from the order Brassicales.

**Results:**

The *S. persica* cp genome was 153,379 bp in length containing a sizeable single-copy region (LSC) of 83,818 bp which separated from the small single-copy region (SSC) of 17,683 bp by two inverted repeats (IRs) each 25,939 bp. Among these genomes, the largest cp genome size (160,600 bp) was found in *M. oleifera,* while in *S. persica* it was the smallest (153,379 bp). The cp genome of *S. persica* encoded 131 genes, including 37 tRNA genes, eight rRNA genes and 86 protein-coding genes. Besides, *S. persica* contains 27 forward, 36 tandem and 19 palindromic repeats. The *S. persica* cp genome had 154 SSRs with the highest number in the LSC region. Complete cp genome comparisons showed an overall high degree of sequence resemblance between *S. persica* and related cp genomes. Some divergence was observed in the intergenic spaces of other species. Phylogenomic analyses of 60 shared genes indicated that *S. persica* formed a single clade with *A. tetracantha* with high bootstrap values. The family Salvadoraceae is closely related to Capparaceae and Petadiplandraceae rather than to Bataceae and Koberliniacaea.

**Conclusion:**

The current genomic datasets provide pivotal genetic resources to determine the phylogenetic relationships, genome evolution and future genetic diversity-related studies of *S. persica* in complex angiosperm families.

**Supplementary Information:**

The online version contains supplementary material available at 10.1186/s12864-021-07626-x.

## Introduction

Salvadoraceae is a small family that comprises three genera, *Salvadora* Juss (±five species), *Azima* Lam. (±four species) and *Dobera* Juss. (two species) [1]. Salvadoraceae contains small trees and shrubs growing in arid environments and widespread worldwide. The main trunk of *S. persica* is erect or trailing and can grow up to 10 m with a circumference of 3 ft. Tree bark is rough with a brownish color and young branches are greenish [2, 3]. *S. persica* showed variation in different countries, which may be due to the climatic conditions, anthropogenic activities and water resources [4]. It is native to Saudi Arabia, Pakistan, Nigeria, Egypt, Uganda Algeria, India, Zimbabwe and Sri Lanka [5]. *S. persica* is a non-deciduous, slow-growing perennial halophyte that can grow under extreme dry and saline conditions [6]. The Arabic name of *S. persica* is Khardal Shajar-el-Miswak. At the same time, in English it is called Mustard tree or Toothbrush tree [7]. For oral hygiene, chewing sticks have been used since 3500 BC by Babylonians. *S. persica* L. is an economically and medicinally plant with numerous medicinal properties. It has been used in traditional medicine, especially in the Middle East and Eastern Africa [4]. Phytochemically, the *S. persica* contains a higher proportion of fluorides. In contrast, it has shown considerable prospects for antimicrobial and anticancer due to the presence of benzyl isothiocyanate, alkaloids, salvadoside and salvadoraside, etc. [8].

Though *S. persica* has been utilized substantially by local communities, taxonomically, the family had suffered a lot due to displacement. It has always been classified as an outsider, dumped in or close to Oleales [9] or Celastrales [10, 11] or either as ‘incerta sedis’ [12]. In the beginning [13], it was placed in an extended order Capparales and later separated the family into distinct order Salvadorales [14]. Using chemical markers, Salvadoraceae was classified early with Capparales (Brassicales) [13, 14] due to custard oil. Later, its association with all mustard oil-producing families was confirmed by various genes phylogeny [15–17]. However, with the advancement in molecular methods, genetic variations have helped solve several taxonomic problems [18]. Up till now, numerous types of molecular markers have been designed, assessed, and categorized into various groups such as polymerase chain reaction (PCR)-based features, simple sequence repeats (SSRs) and inter simple sequence repeats, random amplified polymorphic DNA, single-nucleotide polymorphism, hybridization-based molecular markers, and amplified fragment length polymorphism [19, 20]. Using some of these methods, Salvadoraceae was considered a sister family to Bataceae with strong support. Koeberliniaceae is regarded as a sister to these two families in a clade near core Brassicales. Recent combined molecular and morphological analysis of Brassicales supported this association [21]. Despite their importance of these molecular methods, there are still several disadvantages at certain levels of principles [20]. However, with the current advancements in next-generation sequencing methods and platforms, understanding large-scale genome composition, precisely, chloroplast genome, has shown unprecedented progress in exploring taxonomic and evolutionary challenges to important plant species [22, 23].

The chloroplast is a vital plant organelle in green plants that plays a keycrucialle in plant cells during carbon fixation and photosynthesis [24]. In angiosperms mostly these cp genomes are uniparentally inherited circular DNA molecules ranging from ~ 115 to 165 kb in length [25], and these differences are primarily due to IR contraction/expansion loss [26]. Moreover, in most angiosperms, these genomes are divided into four parts containing one small single-copy (SSC) region, one large single-copy (LSC) region, and two same length inverted repeat regions (IRs) regions [27, 28]. In terms of gene structure and composition, the cp genome is more conserved than the mitochondrial and nuclear genome [29, 30]. Cp genomes are a valuable genetic resource to infer the phylogenetic position of different species due to their highly conserve and non-recombinant nature [31, 32]. Comparatively, it has become an easy and cheap resource to sequence due to recent advancements in next-generation sequencing technology to solve the controversial phylogenetic questions of non-model taxa and infer their phylogenetic position complete cp genome and shared genes [22, 23].

More than 6500 chloroplast genomes are sequenced until now; however, there are still many economically and medicinally important plant species that haves no genomic datasets [33]. Notwithstanding the wide distribution of family Salvadoraceae in arid areas, very little is known about this family genetically. There is no genomic information at the species, genus, or family level. Hence, the current study was aimed to establish genomic datasets for *S. persica* as well for Salvadoraceae. The present study also characterized the whole cp genome of *S. persica* and compared it with the 11 available cp genomes from Brassicales. Furthermore, we performed phylogenomic assessment based on the shared genes amongst the 31 cp genomes from order Brassicales*.*

## Results

### *S. persica* chloroplast genome: composition and structure

**Fig. 1:**
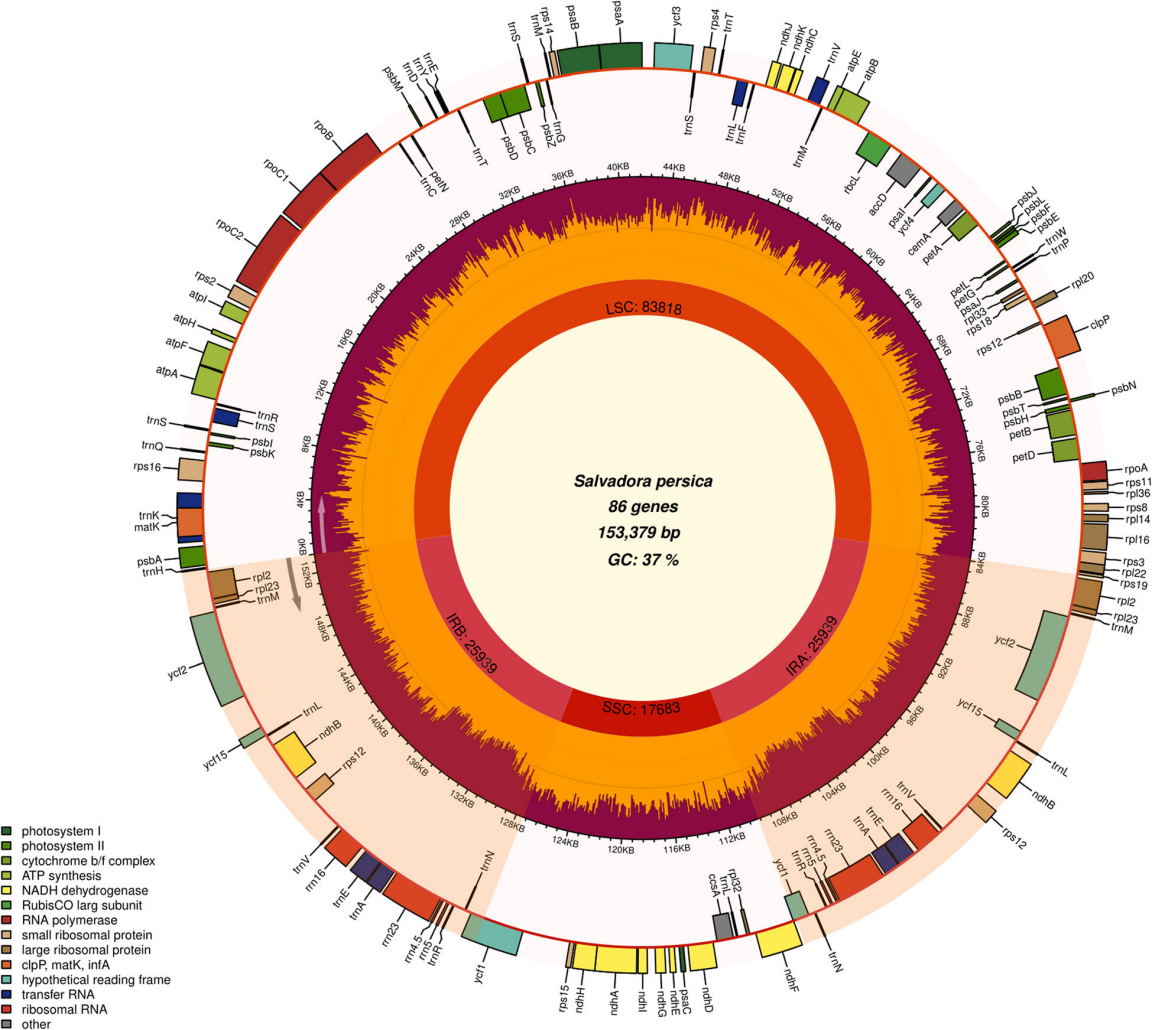
Genomic map of the *S. persica* cp genome. The pink part inside the inner green circle indicates the extent of the inverted repeat regions (IRa and IRb; 25,949 bp), which separate the genome into small (SSC; 17,683 bp) and large (LSC; 83,798 bp) single copy regions. Genes drawn inside the circle are transcribed clockwise, and those outsides are transcribed counter clockwise. Genes belonging to different functional groups are color-coded. The red in the inner circle corresponds to the GC content, and the light green corresponds to the AT content

The assembly and detailed bioinformatic analyses showed that the chloroplast (cp) genome size of *S. persica* is 153,379 bp. It has a distinctive quadripartite structure which consists of LSC (83,818 bp) region, separated from the SSC region (17,683 bp) by two inverted repeats (IRs; 25,939 bp) (Fig. 1; Table 1). The cp genome of *S. persica* comprises 131 genes, including 86 protein-coding genes (9 large and 12 small ribosomal subunits, 43 photosynthesis-related proteins, four DNA-dependent RNA polymerase, and ten genes encoding other proteins), 37 tRNA genes, and eight rRNA genes (Table S1). About 22 genes containing introns were determined in the *S. persica* cp genome, including 12 protein-coding genes and eight tRNA genes (with one intron), whereas the other two protein-coding genes (*ycf3* and *clpP*) with two introns (Table 2). The *matK* gene is present in the intronic region of *trnK*-UUU gene which had the largest intron (2549 bp). Similarly, the *ycf15* gene had the smallest intron (295 bp) (Table 3). The trans-spliced gene small ribosomal protein-12 (*rps12*) is having single intron. Moreover, its five ′ end exon is present in the LSC region, while the three ′ end exon is duplicated in IR region (Fig. 1). Inclusively, the protein-coding, tRNA and rRNA genes contain 47.2, 1.8 and 5.9%, respectively, in the *S. persica* cp genome. Similar to typical angiosperm cp genomes, the GC composition of tRNA (52.8%) and rRNA (55.3%) is the highest, followed by protein-coding genes (37.4%) in the coding regions. Codon – anticodon characteristic pattern and codon usage of *S. persica* cp genome is summarized in Table 3. The most frequent amino acid was leucine (10.8%), whereas the least frequent one was cysteine (1.2%). The GC content of the *S. persica* cp genome is 36.7%, whereas the LSC, SSC, and IR regions’ GC content is 34.6, 30.2, and 42.2%, respectively. Similar results were observed in related species. However, the highest GC contents in the IR regions are due to the high GC contents of eight rRNA genes located in these regions.

**Table 1:** Summary of complete chloroplast genomes

	***S. persica***	***A. tetracantha***	***A. arabicum***	***A. thaliana***	***B. nigra***	***C. rubella***	***C. papaya***	***M. oleifera***	***R. carnosula***	***R. cretica***	***C. limprichtiana***	***T. hassleriana***
**Size (bp)**	153,379	153,415	154,234	154,478	153,633	154,601	160,100	160,600	154,328	154,188	153,746	157,688
**Overall GC contents**	36.7	36.1	36.6	36.3	36.4	36.5	36.9	36.8	36.1	36.3	36	35.8
**LSC size in bp**	83,818	83,841	83,401	84,170	83,552	83,990	88,749	88,563	83,463	83,274	83,293	87,509
**SSC size in bp**	17,683	17,488	17,716	17,780	17,695	17,855	18,701	18,881	18,130	18,169	17,763	18,677
**IR size in bp**	25,939	26,043	26,558	26,264	26,193	26,462	26,325	26,570	26,367	26,372	26,262	25,804
**Protein coding regions size in bp**	72,281	78,288	79,482	77,925	79,881	78,489	78,636	79,881	78,708	76,734	50,010	79,755
**tRNA size in bp**	2811	2784	2789	2791	2790	2792	2792	2739	2826	2826	2623	2863
**rRNA size in bp**	9052	9054	8929	8929	9050	9052	9050	9050	9050	9050	8929	9400
**Number of genes**	131	131	128	129	132	130	131	129	130	132	113	131
**Number of protein coding genes**	86	84	83	85	87	84	84	85	85	85	71	85
**Number of rRNA**	8	8	8	7	8	8	8	8	8	8	7	8
**Number of tRNA**	37	37	37	37	37	37	37	36	37	37	35	38

**Table 2:** The lengths of introns and exons for the splitting genes

Gene	Strand	Start	End	ExonI	IntronI	ExonII	IntronII	ExonIII
*atpF*	–	11,074	12,350	145	722	410		
*petB*	+	74,594	75,997	6	756	642		
*petD*	+	76,196	77,382	8	704	475		
*rps16*	–	4913	6034	40	885	197		
*rpoC1*	–	20,203	23,031	432	786	1611		
*ycf3*	–	42,381	44,403	118	739	230	783	153
*clpP*	–	69,667	71,658	71	834	294	567	226
*rpl2*	–	83,985	85,494	391	685	434		
*ycf15*	+	93,141	93,666	77	295	154		
*ndhB*	–	94,345	96,563	775	686	758		
*ndhB*	+	140,635	142,853	775	686	758		
*ycf15*	–	143,532	144,057	77	295	154		
*rpl2*	+	151,704	153,213	391	685	434		
*ndhA*	–	118,768	120,965	553	1106	539		
*trnS-CGA*	+	8236	9016	31	690	60		
*trnE-UUC*	+	102,180	103,201	32	950	40		
*trnA-UGC*	+	103,265	104,136	37	799	36		
*trnA-UGC*	–	133,062	133,933	37	799	36		
*trnE-UUC*	–	133,997	135,018	32	950	40		
*trnL-UAA*	+	47,067	47,658	35	507	50		
*trnV-UAC*	–	51,131	51,809	39	603	37		
*trnK-UUU*	–	1582	4202	37	2549	35

**Table 3:** Codon Usage in this chloroplast genome

Codon	Amino acid	Frequency	Number
GCA	A	13.735	615
GCC	A	6.968	312
GCG	A	5.65	253
GCT	A	23.628	1058
TGC	C	3.64	163
TGT	C	8.553	383
GAC	D	8.553	383
GAT	D	30.172	1351
GAA	E	37.899	1697
GAG	E	11.993	537
TTC	F	19.966	894
TTT	F	41.227	1846
GGA	G	25.817	1156
GGC	G	6.164	276
GGG	G	11.725	525
GGT	G	22.445	1005
CAC	H	5.874	263
CAT	H	19.452	871
ATA	I	25.415	1138
ATC	I	16.37	733
ATT	I	43.683	1956
AAA	K	39.261	1758
AAG	K	14.74	660
CTA	L	12.819	574
CTC	L	7.348	329
CTG	L	7.727	346
CTT	L	22.489	1007
TTA	L	33.723	1510
TTG	L	22.11	990
ATG	M	22.132	991
AAC	N	11.166	500
AAT	N	37.207	1666
CCA	P	12.082	541
CCC	P	7.839	351
CCG	P	4.757	213
CCT	P	15.41	690
CAA	Q	28.184	1262
CAG	Q	7.281	326
AGA	R	17.107	766
AGG	R	7.258	325
CGA	R	13.668	612
CGC	R	3.864	173
CGG	R	4.288	192
CGT	R	12.73	570
AGC	S	5.516	247
AGT	S	16.035	718
TCA	S	16.191	725
TCC	S	12.149	544
TCG	S	6.812	305
TCT	S	22.512	1008
ACA	T	15.231	682
ACC	T	9.357	419
ACG	T	4.98	223
ACT	T	19.273	863
GTA	V	19.206	860
GTC	V	7.325	328
GTG	V	7.683	344
GTT	V	19.072	854
TGG	W	18.849	844
TAC	Y	7.035	315
TAT	Y	30.596	1370
TAA	*	3.395	152
TAG	*	2.211	99
TGA	*	2.457	110

### Comparative analysis of *S. persica* cp genome with the cp genome of related species

**Fig. 2:**
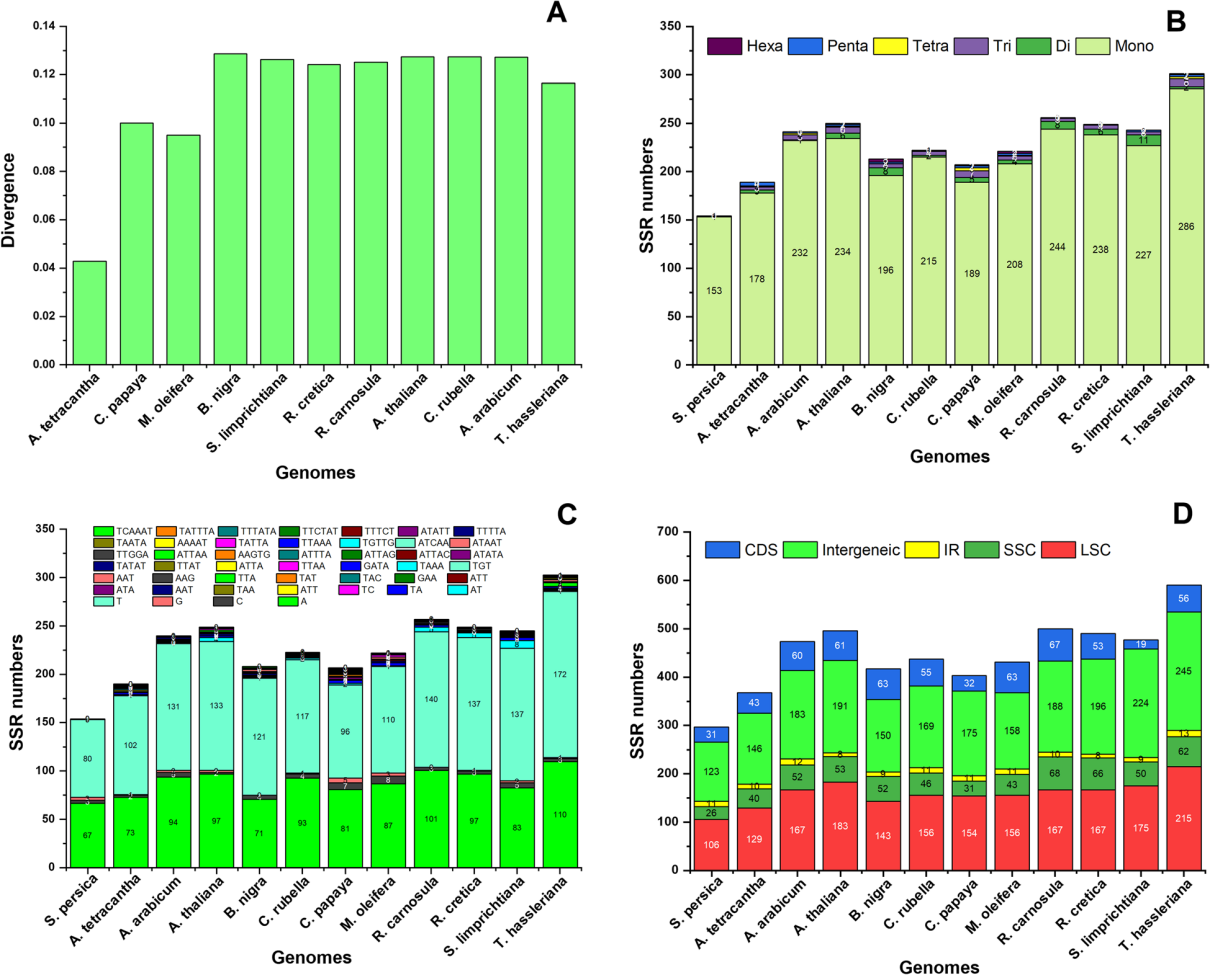
Evolutionary sequence divergence and simple sequence repeats (SSR) Analysis. Estimates of Evolutionary Divergence among *S. persica* and related cp genomes (**a**) Analysis of simple sequence repeats in the twelve chloroplast genomes including *S. persica* (**b**), frequency of identified SSR motifs in different repeat class types (**c**), SSR numbers detected in the twelve species LSC, SSC, IR, CDS and Intergenic regions (**d**)

The *S. persica* cp genome was compared with other eleven cp genomes (*A. tetracantha, A. arabicum, A. thaliana, B. nigra, C. rubella, C. papaya, M. oleifera, R. carnosula, R. cretica, C. limprichtiana* and *T. hassleriana*) from six families Salvadoraceae, Apocynaceae, Brassicaceae, Caricacrea, Moringaceare, and Cleomaceae. The results revealed that the genome size of *M. oleifera* (160,600 bp) is the largest of these, followed by *C. papaya* (160,100 bp). In comparison, the smallest genome sizes were detected in *S. persica* (153,379 bp) and *A. tetracantha* (153,415 bp) from family Salvadoraceae. This difference in size was accredited to the LSC region’s size (Table 1). Analysis of genes with known function revealed that *S. persica* shared 71 genes with other 11 species cp genomes. The highest number of protein coding genes (PCGs) were detected in *B. nigra* (87) while lowest were observed in *C. limprichtiana* (71) (Table 1). Overall, the current results are showing a high rate of sequence resemblances among protein-coding and IR region (Figure S1). However, maximum amount of sequence divergences was observed in many intergenic regions, especially *atpH – atpI, trnK-rps16, trnT-pscbD, rpoB-trnC, rps4-ndhJ, petA-psbL, rbcL-accD, ndhC-trnV* and *ycf4-cemA*. Similarly, some divergences were also observed in protein-coding genes, including *ycf1, rpl16, clpP, rpoC1, rpoC2, ndhA, atpF, ndhF* and *ycf15* (Figure S1). In pairwise sequence divergences, *S. persica* showed maximum divergences (0.28) with *B. nigra* and lowest with *A. tetracantha* (0.042) (Fig. 2a). Moreover, many SNP and InDel substitutions were revealed in the *S. persica* cp genome coding region and related species. The highest number of InDels were detected in *T. hassleriana* (352), while the lowest was observed in *B. nigra* (6). On the other hand, highest number of SNPs was detected in *T. hassleriana* (9935) and the lowest was detected in *B. nigra* (1009) (Table S2).

### Microsatellite markers arrangement in cp genome

In microsatellite analysis, a considerable variation was observed in order Brassicales. The lowest number of SSRs were detected in *S. persica* (154) and *A. tetracantha* (189) from the family Salvadoraceae. Similarly, *T. hassleriana* having highest microsatellite repeats, i.e., 301 followed by *R. carnosula* (256) and *A. thaliana* (250) (Fig. 2b). In *S. persica* cp genome about 153 SSRs were mononucleotide while one SSR is dinucleotide. Similarly, in *A. tetracantha* 178 mononucleotides, three di, three tri, and one tetra, four pentanucleotides were found. The hexanucleotide was absent in this genome. *A. arabicum’* genome contained 232 mono, one di, five tri, two tetra and one pentanucleotide. *A. thaliana* has 234 mononucleotides, six di, six tri, one tetra, two Penta, and one hexanucleotide. *B. nigra* contain 196 mononucleotides, eight di, four tri, two penta and three hexanucleotide while tetranucleotide is absent. *C. rubella* has 215 mononucleotides, two di, four tri and one tetranucleotide, penta and hexanucleotide are missing here (Fig. 2b). Furthermore, mononucleotides are most abundant nucleotides among all six types of nucleotides in all cp genomes. In *S. persica*, almost 52.8% of the mononucleotide contain a T motif and 43.7 have A motif. A comparable pattern of SSR-motif was noted in related cp genomes (Fig. 2c). Among these SSRs 31 and 43 SSRs were found in coding-regions of *S. persica* and *A. tetracantha,* respectively. Similarly, in *S. persica* 106, 26, 11 and 123 SSRs were identified in LSC, SSC, IR and non-coding regions, respectively (Fig. 2d).

### Repeat distribution in *S. persica* cp genome

**Fig. 3:**
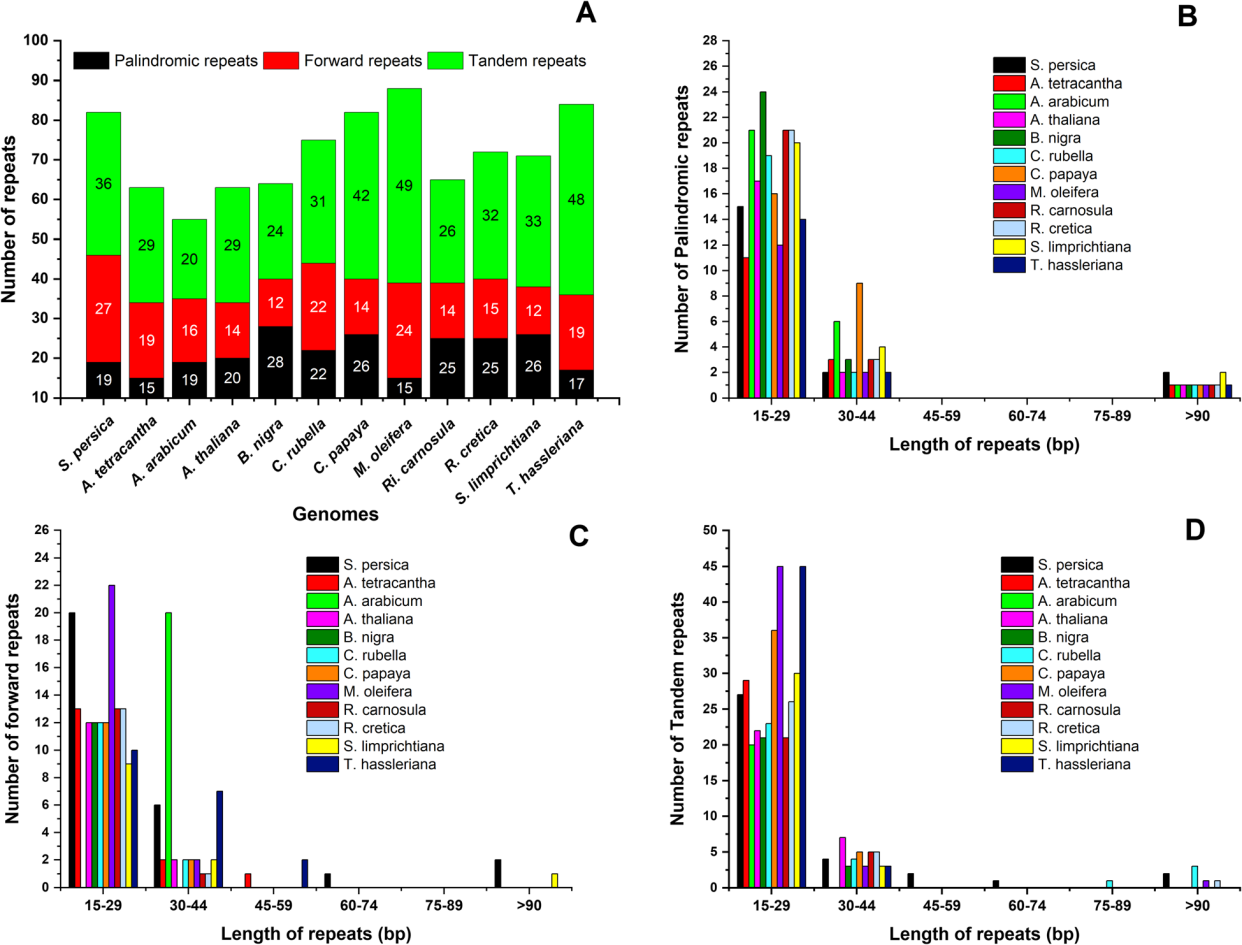
Analysis of repeated sequences in *S. persica* and other eleven cp genomes. **a** Totals numbers of three repeat types; **b** Number of palindromic repeats by length; **c** Number of forward repeats by length; **d** Number of tandem repeats by length

In the current study, we studied different repeat sequences i.e., palindromic, forward and tandem repeats in *S. persica* chloroplast genome and compared it with 11 others cp genome genomes (Fig. 3). The results showed that *S. persica* contains 19 palindromic, 27 forward and 36 tandem repeats. *A. tetracantha* had 15 palindromic, 19 forward and 29 tandem repeats (Fig. 3). In *S. persica* repeats, 15 palindromic repeats were 15–29 bp, 2 were 30–44 bp in length while 2 were > 90 bp in length. In the case of forward repeats, 20 repeats were 15–29 bp, six repeats were 30–44 bp,1 was 60–74 bp in length, and 2 were > 90 bp in length. Similarly, 27 tandem repeats were15–29 bp in length, 4 were 30–44 bp in length, 2 were 45–59 bp in length, one repeat was 60–74 bp in length and 2 were > 90 bp in length. Furthermore, among these cp genomes, highest number of tandem repeats were detected in *M. oleifera* (49) followed by *C. limprichtiana* (48), while the lowest number was detected in *A. arabicum* (20). Similarly, the highest number of forward repeats were detected in *S. persica* (27), while lowest was seen in *B. nigra* and *C. limprichtiana* (12). However, the highest number of palindromic repeats were in *B. nigra* (28) (Fig. 3).

### IR expansion and contraction in *S. persica* cp genome

**Fig. 4:**
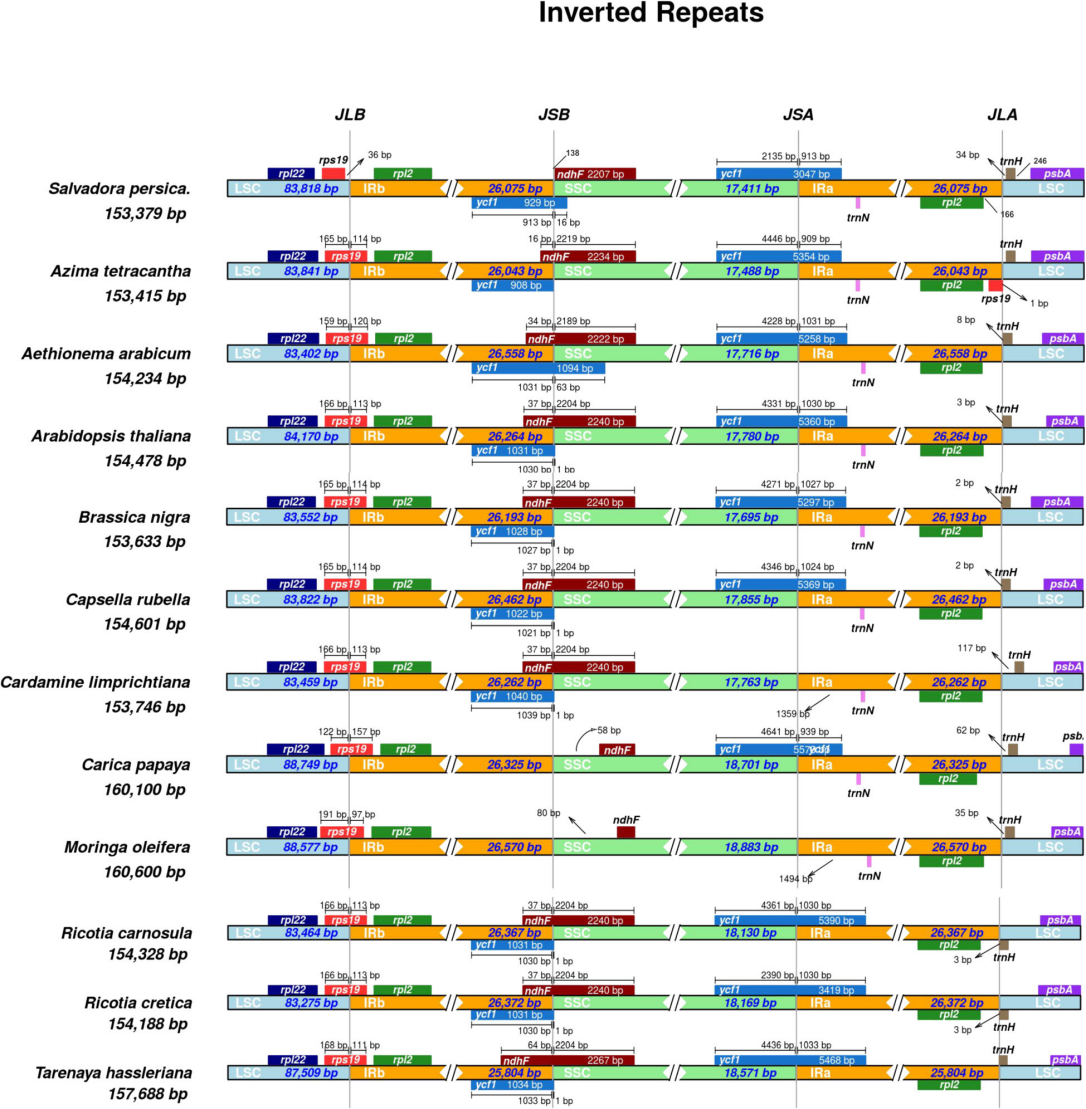
Distance between adjacent genes and junctions of the small single-copy (SSC), large single-copy (LSC), and two inverted repeats (IR) regions of *S. persica* with related species cp genomes. Boxes above and below the mainline indicate the adjacent border genes. The figure does not scale regarding sequence length and only shows relative changes at or near the IR/SC borders

In most angiosperms cp genomes IR regions are reported to be the most conserved regions. The larger IR length correlates with larger cp genome sizes. The IR length in *S. persica* is similar to previously reported angiosperm genomes. In the present study, comparative assessment of 4 junctions viz. JSA, JSB, JLA and JLB with IRa and IRb, two single copy regions, and *S. persica* with related 11 related species were performed (Fig. 4). Despite the similar lengths of *S. persica* and related genomes, some enlargement and shrinkage were noted within the IR region, ranging from 25,804 bp in *T. hassleriana* to 26,570 bp in *M. oleifera*. Results revealed that in *S. persica* the *rps19* gene present 36 bp away from JLB junction toward the LSC region. The *rpl2* gene occupied IRB region, the *ycf1* gene overlapped the JSB junction and 913 bp present in IRB and 16 bp in SSC region. The *ndhF* gene occupied the SSC region about 138 bp away from JSB border and the *trnN* present in IRA region, while *trnH* present 34 bp away from JLA junction toward LSC region (Fig. 4).

Similarly, the *psbA* gene is resent in the LSC region. On the other hand, in A. *tetracantha* the *rpl22* gene present in LSC region, the *rps19* gene present across the JLB junction 165 bp toward LSC region and 114 bp toward IRB, *ycf1* gene present in IRB region while *ndhF* present 16 bp in IRB region and 2234 in SSC region while the *trnN* gene present in IRA region. The *rps19* gene in all other cp genomes showed almost similar result like *A. tetracantha*. In the case of *ycf1* gene, the locations were also the same as *A. tetracantha* except *A. arabicum* in which the gene present across the JSB junction and in *C. papaya* present across JSA junction while in *M. oleifera* it is absent. The *ndhF* gene in *C. papaya* and *M. oleifera* 58 and 80 bp away from JSB junction toward the SSC side. The *trnN* gene occurs in the same position in all genomes but was absent in *R. carnosula*, *R. cretica* and *T. hassleriana.* The *trnH* occurs 117 bp away from JLA junction toward the LSC region.

### Phylogenomic assessment of *S. persica*

**Fig. 5:**
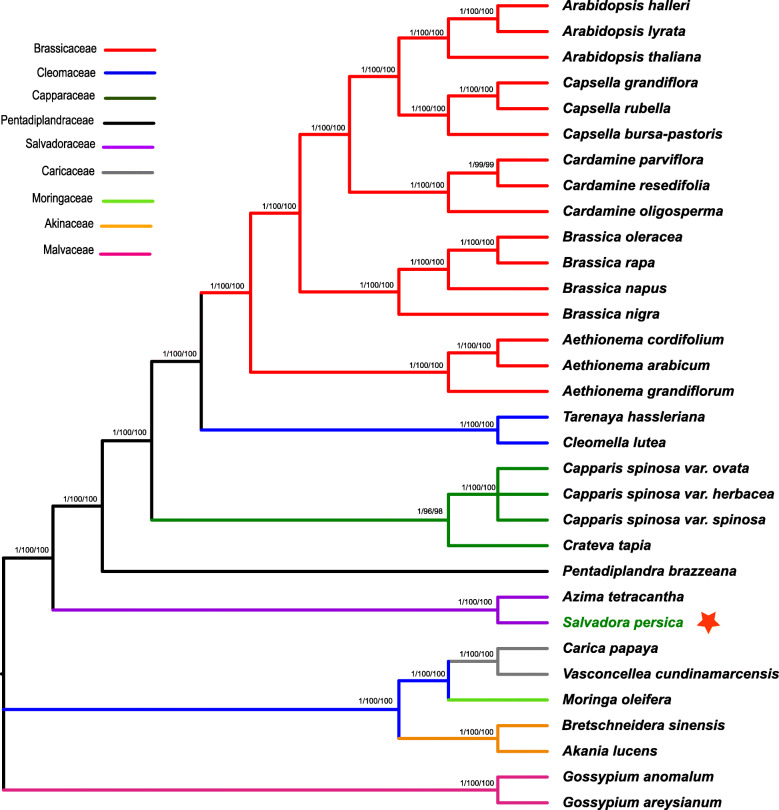
Phylogenetic trees of *S. persica* with related 31 species from class Brassicales. The 60 shared genes dataset was analyzed using Bayesian inference (BI), maximum parsimony (MP), and maximum likelihood (ML). Numbers above the branches represent bootstrap values in the MP and ML. The red star represents the position of *S. persica*

In the current study, the phylogenomic disposition of *S. persica* within the order Brassicales was revealed by analyzing multiple alignments of 60 shared genes from 9 families representing 32 genera (Fig. 5). The overall concatenated alignment size from the 60 protein – coding genes was 63,045 bp. *Gossypium anomalum* and *G. areysianum* species were set as the outgroup. Phylogenetic analysis using Maximum parsimony (MP), Bayesian inference (BI), and Maximum likelihood (ML) were performed. The results revealed that *S. persica* forms a single-clade with *A. tetracantha* showing highest bootstrap values. Similarly, this study also revealed that the family Salvadoraceae is closely related to Caricaceae, Petadiplandraceae, and Capparaceae.

## Discussion

The current study showed the first complete cp genome sequence for *S. persica*, genus *Salvadora* and family Salvadoraceae. Further, the cp genome was compared with eleven cp genomes of related species from order Brassicales. These cp genomes ranged from 153 kb to 160 kb in size and comprised all the four major components of chloroplast genome architecture. All the cp genomes are conserved and in the same range, and its genome sizes ranging from 153,379 bp in *S. persica* to 160, 600 bp in *M. oleifera*, which encoded 113–132 genes (131 in *S. persica*, 132 in *B. nigra* and 113 in *C. limprichtiana*) (Table 1). The size range of *S. persica cp* genome is found in the same range as the sizes of the previously reported cp genomes of *A. tetracantha* (153,415 bp) and other related species [29, 34–36]. Like typical angiosperms cp genomes (20 ± 28 kb), these species’ IRs length ranges from 25 to 26 kb in length [34, 37]. However, some variations were observed in these cp genomes, mainly due to variation in the LSC regions rather than contraction and expansion of IR region, as was found recently [27, 34, 38]. Like other reported cp genomes from Brassicales about 18 genes are duplicated in the IR regions, containing, four rRNA genes, seven tRNA genes, and eight protein-coding genes (PCGs) [39–41].

Furthermore, 22 (eight tRNA genes and 12 protein-coding genes) having introns were determined in these genomes and among these introns containing genes *clpP, ycf3* and *rps12* genes have two introns each (Table 2). In synergy with previously reported cp genomes, angiosperm *rps12* was unequally divided. The maturase K (*matK*) gene is marked within the *trnK* intron as reported in other cp genomes from family Brassicales [42–44]. The *S. persica* LSC, SSC, and IR region’s GC content were 34.6, 30.2, and 42.2%. Like other angiosperm cp genomes, higher GC content was detected in IRs due to 8 rRNAs in these regions [23, 27, 45].

The complete *S. persica* cp genome was compared with the related 11 plant cp genomes. Chloroplast gene analysis with known function revealed that *S. persica* shared 74 protein-coding genes with related species. Furthermore, the gene contents and organization of *S. persica* were similar to those of other Brassicales cp genomes [34, 41, 43]. Similarly, the average pairwise sequence divergence among the *S. persica* and related species’ cp genomes was determined (Fig. 2a). The cp genome of *S. persica* exhibited an average sequence divergence of 0.112 with all species.

In contrast, the highest sequence divergence with *B. nigra* (0.128) whereas the lowest was observed with *A. tetracantha* (0.042). Despite the conserved gene order reported in most plants, some distinguished changes such as sequence inversion [32], gene loss [46] and contraction and expansion at the borders between IRs, SSC, and LSC regions [47, 48]. Similar length variation was observed previously among cp genomes due to the expansion and contraction of the IR regions [48, 49]. The *S. persica* cp genome was highly conservative in structure, size, IR and SC boundary locations. However, due to the contraction and expansion of IR regions some diversion was observed in most land plants [43, 50–53].

A detailed analysis of JSA, JSB, JLA and JLB between two IRs and LSCs of *S. persica* and with 11 related species (A*. tetracantha, A. arabicum, A. thaliana, B. nigra, C. rubella, C. papaya, M. oleifera, R. carnosula, R. cretica, C. limprichtiana,* and *T. hassleriana*) were performed. Despite similar lengths of the IR regions of *S. persica* and related species, some contraction and extension were determined, with the IR regions ranging from 25,804 bp in *T. hassleriana* to 26,570 bp in *M. oleifera*. Despite the four conserved junctions in these cp genomes, some variations were observed with *C. papaya* and *M. oleifera* cp genomes. The *rps19* gene is present 36 bp away in the LSC region in *S. persica*. Simultaneously, in other genomes it is partially duplicated genes detected in the IRs, including 114 bp of *rps19* in *A. tetracantha* (Fig. 4). Previous reports suggested that repeats, playing a pivotal role in cp genome rearrangements, are essential in performing phylogenetic assessments [54, 55]. Also, comparative evaluation of cp genomes has shown that repeat sequences induce indels and substitutions [56], and re-arrangements of cp sequences and their variatihat occur due to improper recombination and slipped strand mispairing of such repeat sequences [54, 57, 58]. The detection of repeat sequences shows that loci are hotspots for genome re-configuration [55, 59], and repeats can be used to proposed molecular markers for population and phylogenetic studies [55].

As reported by various researchers, repeat sequences which can be very useful in phylogenetic studies can contribute significantly to genome rearrangement [60]. Total 82 repeats were noted in the *S. persica* cp genome. Similarly, about 63 repeats were detected in the *A. tetracantha* cp genome. In comparative analysis, higher repeats (88) were found in *M. oleifera* while the lowest was seen in *A. arabcum* (55) cp genome respectively (Fig. 3). In our study, tandem repeats were detected to be the most plentiful in the *M. oleifera* (49) cp genome, showing similar traits to the previously reported cp genome [61, 62]. SSRs are helpful molecular markers to determine a high degree of variation with similar species and have been used to explore population genetics and polymorphisms [63]. SSRs distinguish potentially valuable markers because of maternal inheritance, relative lack of recombination, and their haploid nature for phylogenetic studies [64]. SSRs have been primarily used to analyze gene flow, genetic variation estimation, and analyze the populations’ history animals and plants [65, 66]. We have detected 154 microsatellites in the *S. persica* cp genome and about 123 were observed in non-coding regions. It has been in synergy with angiosperm cp genomes where a higher number of SSRs were revealed primarily on non-coding regions. Approximately, 154, 189, 243. 252, 213, 224, 207, 221, 255, 249, 243 and 301 SSRs were detected in *A. tetracantha, A. arabicum, A. thaliana, B. nigra, C. rubella, C. papaya, M. oleifera, R. carnosula, R. cretica, C. limprichtiana,* and *T. hassleriana* cp genomes, respectively (Fig. 2). Mono SSRs mainly were detected in *S. persica* cp genome. A similar pattern was also reported previously in angiosperms cp genomes [42, 66–68]. Current results are in accordant to recent studies exhibit that the SSRs detected in the cp genome are usually composed of polyadenine or polythymine repeats and rarely comprise tandem guanine (G) and cytosine (C) repeats [56]. Therefore, SSRs extend a greater contribution to the ‘AT’ diversity of *S. persica* cp genomes, as previously reported for different species [37, 69]. These analyses also revealed that approximately 80% of SSRs were determined in non-coding regions. Similar results were reported earlier, showing SSRs are unequally distributed and might give more information to select molecular markers for both intra and inter-specific polymorphisms [70, 71]. Our findings are parallel with other reports from family Brassicaceae that SSRs having ‘A’ or ‘T’ mononucleotide repeats dominated the cp genomes. Furthermore, mono-nucleotide, penta-nucleotide and hexa-nucleotide repeats contained ‘A’ or ‘T’ at higher amount, which revealed a biased base composition, with an overall ‘AT’ richness in the cp genomes [27, 72].

Chloroplast genomes are valuable sources for molecular, evolutionary and phylogenetic studies. In the recent decade, numerous analyses on the comparison of plastid protein-coding genes [73–75] and complete genome sequences [34, 76] have been done to answer the phylogenetic disposition at deep-nodes and improve the mysterious evolutionary relatedness among angiosperms. In this study, the phylogenetic position of *S. persica* within the order Brassicales was established by analyzing multiple alignments of 60 shared genes from 9 families representing 26 genera (Fig. 5). The results revealed that *S. persica* forms a single clade with *A. tetracantha* with high bootstrap and BI through different methods. Similarly, this study also revealed that family Salvadoraceae is affiliated with Caricaceae, Petadiplandraceae and Capparaceae [77]. reported that Salvadoraceae is affiliated in Brassicales based on *trnL-F* as currently considered by most angiosperms systematic.

Similarly, the previous phylogeny based on 18S locus showed association of Salvadoraceae in Brassicales [17, 78]. However, previously based on comparative analysis of floral and seed anatomy and molecular systematic Salvadoraceae, sister to Bataceae and Koberliniacaea near Brassicales. These conflicting findings need to be further analyzed based on complete cp genomes and shared concatenated genes from all representative species. This study is the first cp genome based phylogenetic assessment of genus family Salvadoracee. Therefore, it is necessary to use more species from the family Salvadoraceae and other Brassicales families to understand phylogeny and evolution better.

## Conclusion

In this study, we elucidated the complete chloroplast genome of *S. persica* for the first time. The gene order and cp genome rearrangement of *S. persica* were similar to that of cp genomes of other related species in the order Brassicales. SSRs and repetitive sequences were analyzed in these cp genomes and highest number of SSRs and repeats were detected in *T. hassleriana* and *M. oleifera* respectively. Overall, a high degree of sequence similarity between *S. persica* and related cp genomes was detected. However, some divergence is detected in intergenic regions and some protein coding genes. The results revealed that *S. persica* form a single clade with *A. tetracantha* and the family *Salvadoraceae* is related to Petadiplandraceae and Capparaceae base on 60 cp shared genes. The current study provides a valuable set of information, which could help species identification and facilitate species identification and solve taxonomic questions.

## Methodology

### *S. persica* DNA extraction, sequencing and assembly

*S. persica* young leaves were collected from Jabal Al-Akhdar, Oman (23° 6′ 12.0780″ N; 57° 22′ 47.7984″ E). The voucher specimen (UoN-H101) was deposited in the Herbarium Center, University of Nizwa, Oman after identifying it from Taxonomist (Saif Al-Hathmi) at Oman Botanic Garden, Muscat Oman. Permission (6210/10/73) to collect plants for research purpose was obtained from Ministry of Environment & Climate Affairs, Muscat Oman. The leaf samples were ground into a fine powder with the help of liquid nitrogen. Cp DNA was extracted according to the protocol of [22, 23]. Cp DNA was further cleaned up using DNAeasy Plant Mini Kit (Qiagen, Valencia, CA) by following manufacture protocol. Similarly, genomic libraries were prepared for Ion S5 sequencing (Life Technologies USA, Eugene, OR, USA) by following manufacturer’s instructions. Cp DNA was fragmented into 400 bp enzymatically using Ion-Shear™ Plus Reagents kit and preparing libraries Ion-Xpress™ Plus gDNA Fragment Library kit. These libraries were quantified using Qubit 3.0 and bioanalyzer (Agilent 2100 Bioanalyzer system, Life Technologies USA). The excellent quality library was amplified using the Ion OneTouch™ 2 instrument, and then the Ion OneTouch™ ES enrichment system was used to enrich these amplified libraries. Then the sample was loaded on Ion S5 530 Chip for sequencing by following Ion S5 sequencing protocol.

### *S. persica* chloroplast assembly and genome annotation

A total of 5,526,428 raw reads were produced for *S. persica*. The generated cp genome reads were de novo assembled and then mapped to *A*. *tetracantha,* which was used as reference genome with the help of Bowtie2 assembler (v.2.2.3) [79] in Geneious prime (v.10.2.3) software [80]. CpGAVAS2 [81] was used for *S. persica* cp genome cp genome annotation. To check the annotation results manually BLAST (v.2.8.1) and DOGMA was used [82]. A genomic map was generated by software Chloroplast [83] and inverted repeat sequences were identified through REPuter [84]. For tRNA detection tRNAscan-SE version 1.21 [85] was used. Moreover, for manual adjustment, tRNAscan-SE and Geneious Prime were used to compare and manually adjusted the start, stop codons and intron boundaries with already reported cp genome. Furthermore, mVISTA version 2.1 [86] in Shuffle-LAGAN mode was used for *S. persica* cp genome divergence with related eleven species where *S. persica* was selected as reference genome. The cp genome was submitted to NCBI gene bank and publicly available with accession number MW233589.

### Repeat analysis in *S. persica* cp genome

For reverse and forward repeats identification online software REPuter software [84], was used. About 90% of identities and 15 bp sequences were considered a minimum criterion. Similarly, to detect SSRs MISA software [87] was used with the following search criteria: for mononucleotide repeats ≥10 repeat units; for dinucleotide repeats ≥8 repeat units, for tri and tetra nucleotide repeats ≥4 repeat units; and for penta and hexa nucleotide repeats ≥3 repeat units. Furthermore, tandem repeat Finder version 4.07 [88], with default settings, was used to calculate tandem repeats in these cp genomes.

### *S. persica* phylogenetic analysis and cp genome divergence

Chloroplast genome divergence among *S. persica* and 11 species from order Brassicales were calculated. A comparative analysis method was used to compare gene order and detect the unclear and absent gene annotation after multiple sequence alignments. MAFFT version 7.222 [89], was used to align the complete cp genome and Kimura’s two-parameter (K2P) model [90] was applied to calculate pairwise-sequence divergence. Similarly, to determine the phylogenetic positions of *S. persica* within the class Brassicales was established by downloading 31 cp genome sequences representing 9 genera from the NCBI database. Based on 60 shared genes among these 32 genomes three different approaches were applied to infer phylogenetic tree: maximum parsimony (MP), using PAUP 4.0 [91]; Bayesian inference (BI), implemented in Mr. Bayes 3.1.2 [92] and maximum likelihood (ML) using MEGA 6 [93], using previously described settings [22, 94]. The best substitution model GTR + G was tested by jModel Test version v2.1.02100 according to the Akaike information criterion (AIC) for Bayesian posterior probabilities (PP) in BI analyses. The Markov Chain Monte Carlo (MCMC) method was run using four incrementally heated chains across 1,000,000 generations, starting from random trees and sampling 1 out of every 100 generations. ML analysis parameters were optimized using a BIONJ tree101 as the starting tree with 1000 bootstrap replicates by employing the Kimura 2-parameter model with invariant sites and gamma-distributed rate heterogeneity. Similarly, to estimate the posterior probabilities, the values of first 30% of trees were discarded as burn-in. Maximum parsimony run was based on a heuristic search with 1000 random addition of sequence replicates with the tree-bisection-reconnection (TBR) branch-swapping tree search criterion.

## Supplementary Information

**Additional file 1: Figure S1.** Visual alignment of chloroplast genomes from *S. persica* with related 11 chloroplast genomes from order Brassicales. VISTA-based identity plot showing sequence identity among 11 species, using *S. persica* as a reference. The vertical scale indicates percent identity, ranging from 50 to 100%. The horizontal axis indicates the coordinates within the chloroplast genome. Arrows indicate the annotated genes and their transcription direction. The thick black lines show the inverted repeats (IRs).

**Additional file 2: Table S1.** Gene composition in this chloroplast genome.

**Additional file 3: Table S2**. SNP and Indel analysis of *S. persica* cp genome with related 11 species.

## Data Availability

The datasets generated during the current study are available in the NCBI GenBank ((https://www.ncbi.nlm.nih.gov; Accession number MW233589).
