# Supplementary figures and images for: Decoding first complete chloroplast genome of toothbrush tree (Salvadora persica L.): insight into genome evolution, sequence divergence and phylogenetic relationship within Brassicales
Source: BMC Genomics. 2021 Apr 30;22:312. doi: 10.1186/s12864-021-07626-x (PMC8086069; doi:10.1186/s12864-021-07626-x)

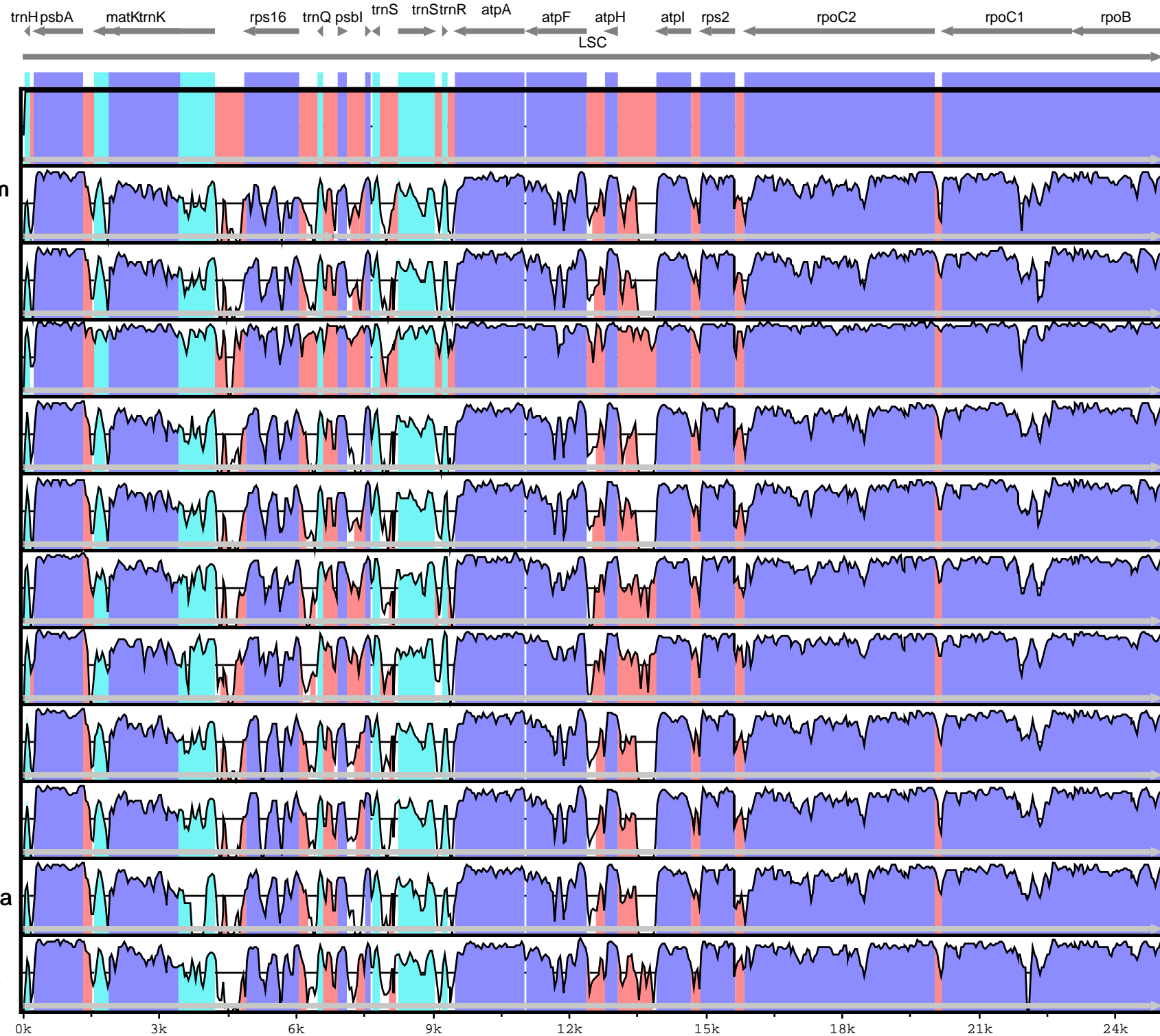

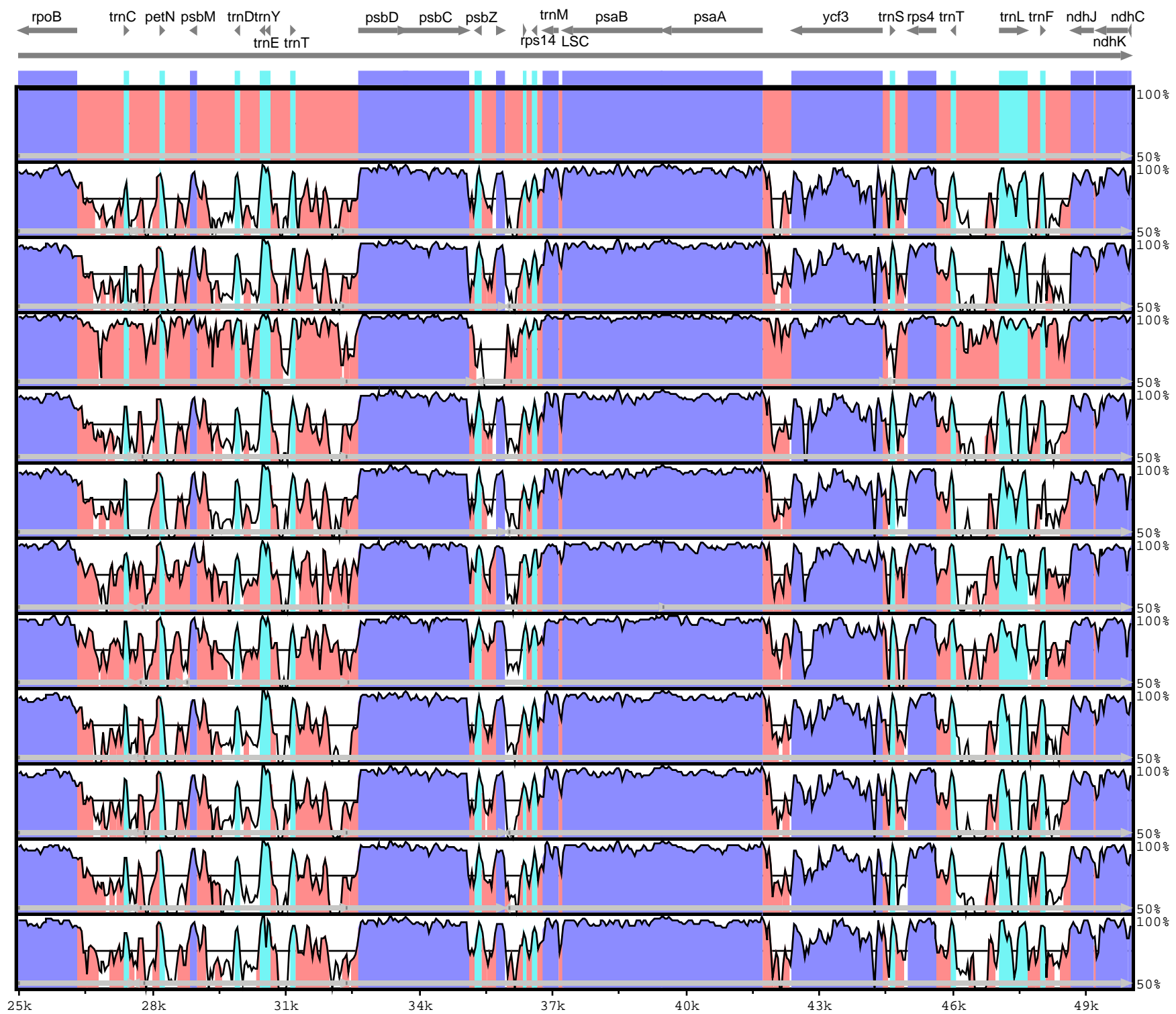

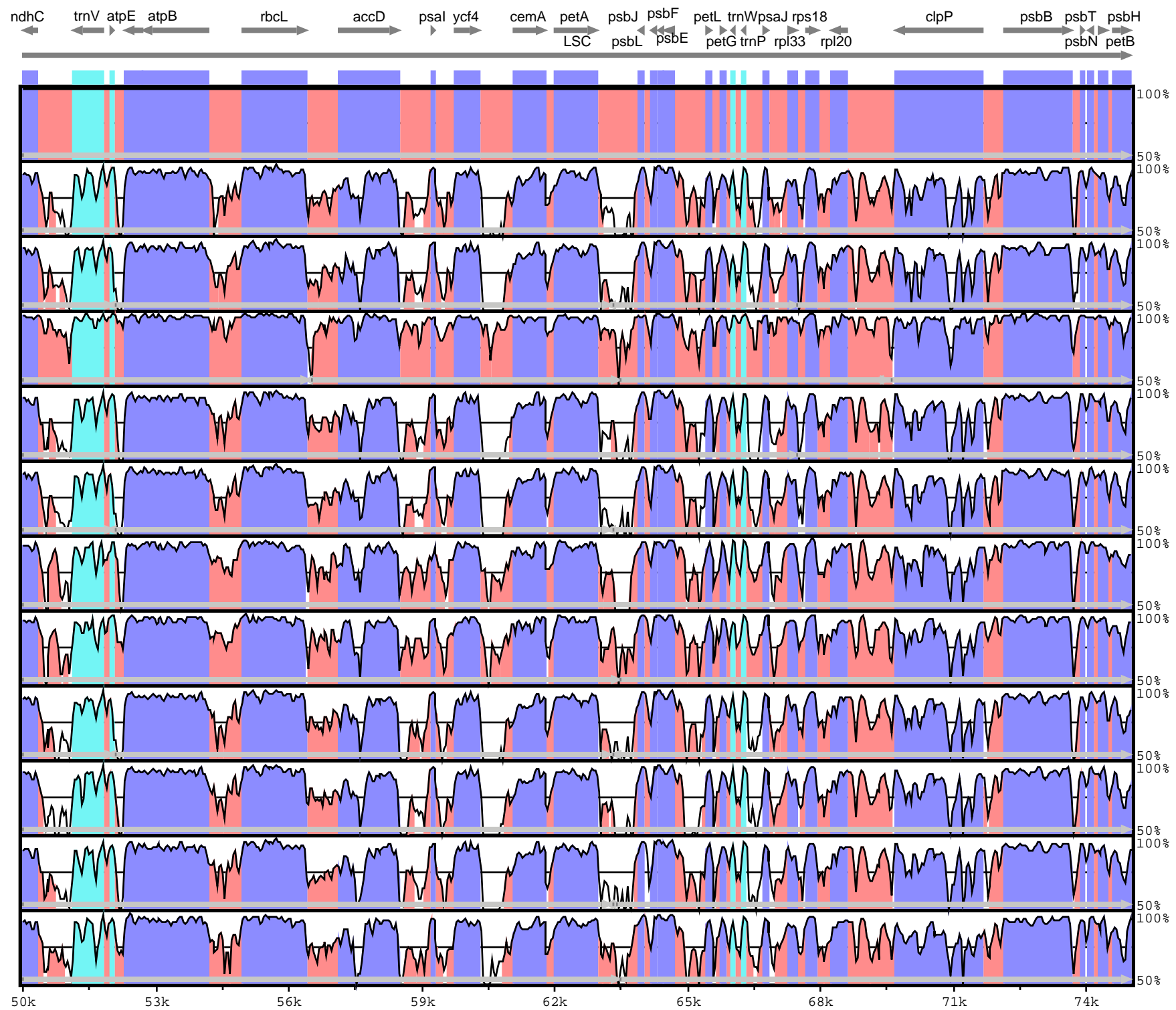

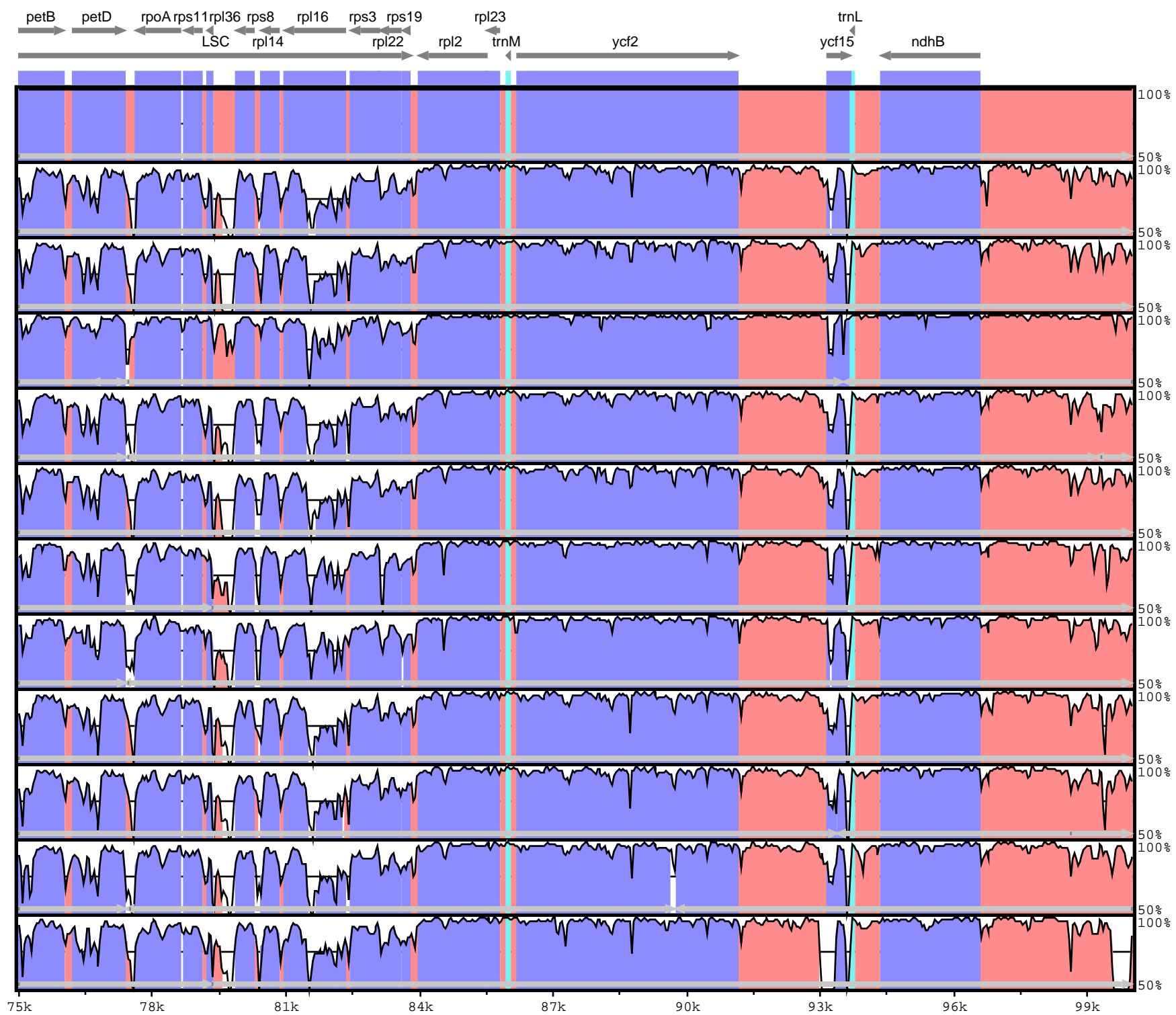

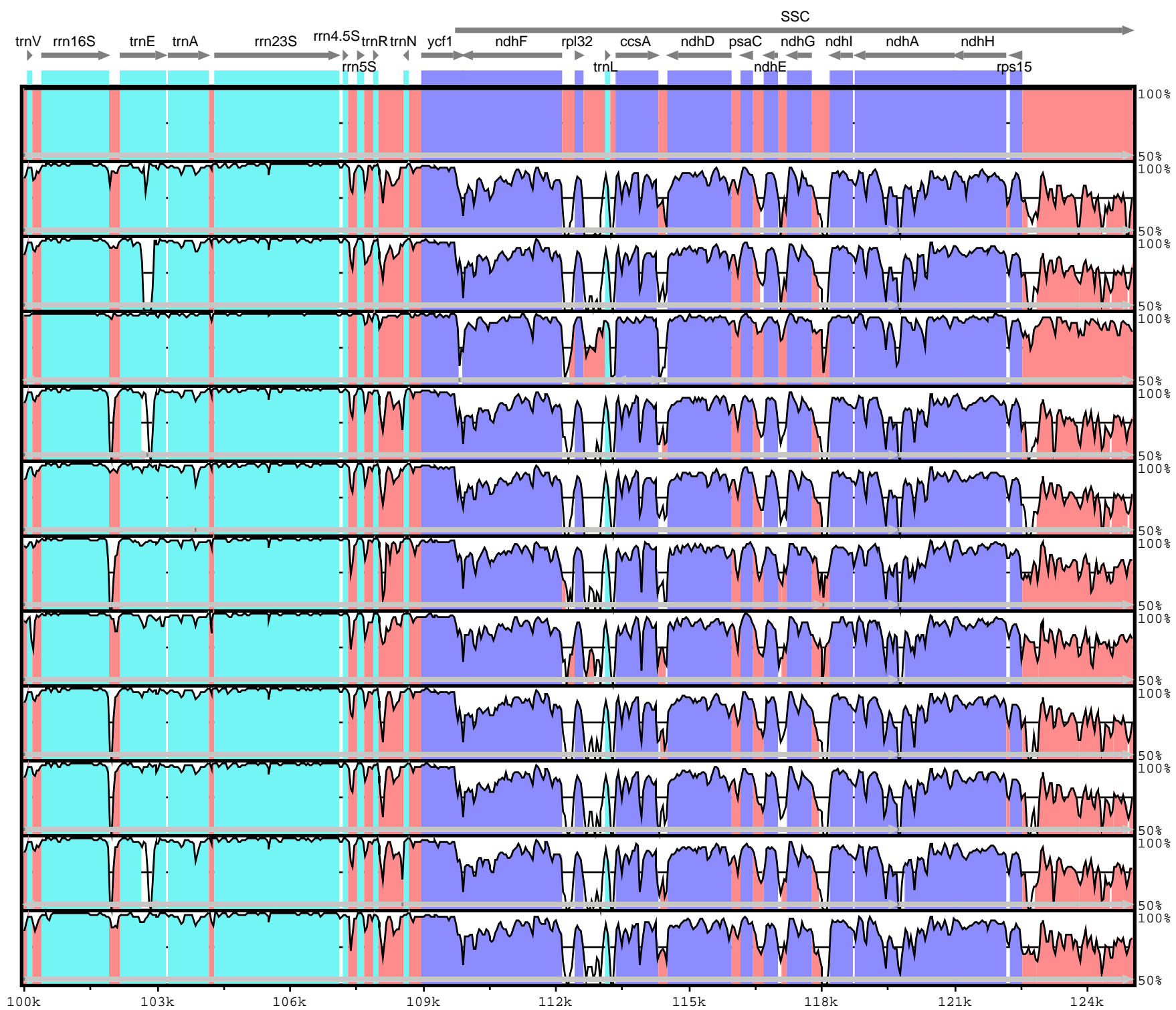

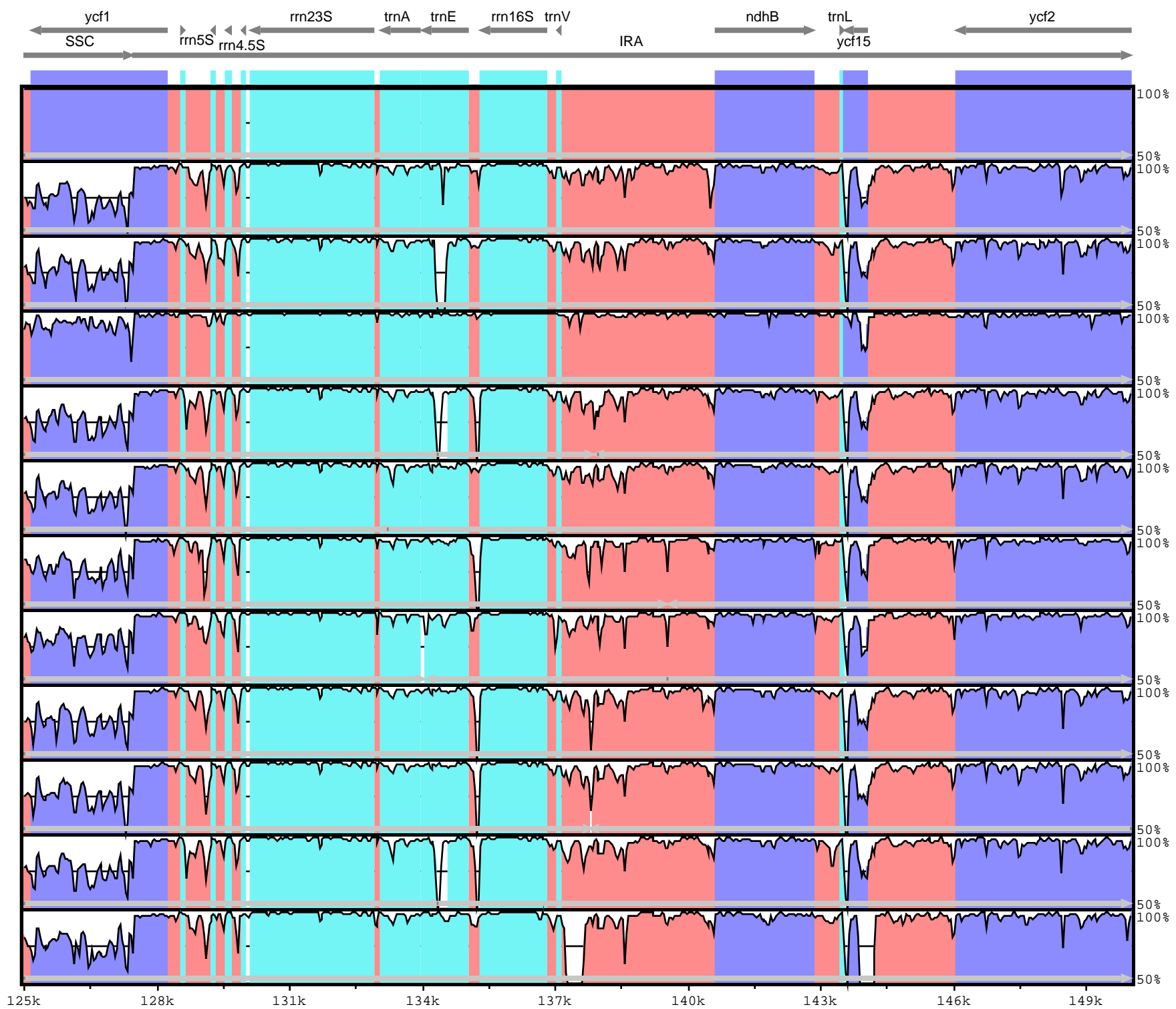

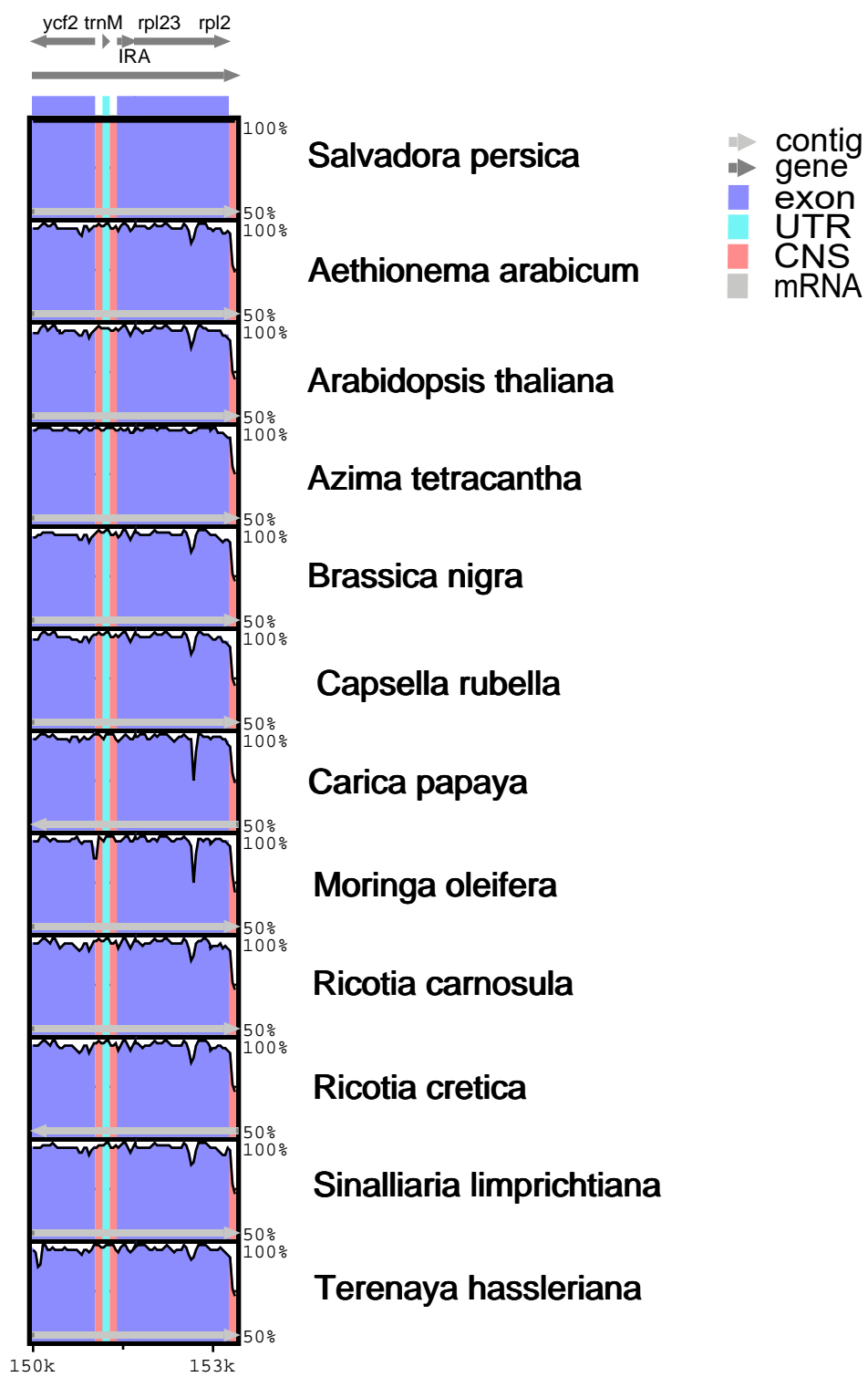

Supplement: Supplementary file 1 — Additional file 1: Figure S1. Visual alignment of chloroplast genomes from S. persica with related 11 chloroplast genomes from order Brassicales. VISTA-based identity plot showing sequence identity among 11 species, using S. persica as a reference. The vertical scale indicates percent identity, ranging from 50 to 100%. The horizontal axis indicates the coordinates within the chloroplast genome. Arrows indicate the annotated genes and their transcription direction. The thick black lines show the inverted repeats (IRs). [file 12864_2021_7626_MOESM1_ESM.pdf]
